# Subjective and Objective Measures of Family Dynamics Within Multigenerational Late-Life Families

**DOI:** 10.1093/geroni/igaf122.3308

**Published:** 2025-12-31

**Authors:** Duyen Tran, Yichen Zhang, Brian Carpenter

**Affiliations:** Washington University in St. Louis, St. Louis, Missouri, United States; Washington University in St. Louis, St. Louis, Missouri, United States; Washington University, St. Louis, Missouri, United States

## Abstract

Late-life families are complex systems that play a significant role in older adults’ lives, making it essential to understand intergenerational relationships. Traditional measures of family dynamics have relied on reports from just one person in the family and on self-report scales, which are vulnerable to potential biases. To address these issues, this study compared reports from both older parents and adult children on the Family Adaptability and Cohesion Scale IV and examined associations with performance on a triadic problem-solving task. Participants were 32 families, consisting of an older parent (M age = 75; 93% female) and two adult children (M age = 46; 65% female). Results indicated considerable variability in intergenerational concordance about family features. The mean difference across family members was 2.60 for Cohesion (range = -39.33 to + 37.67, SD = 19.85) and 3.44 for Flexibility (range = -38.17 to + 30.50, SD = 15.33). Across all families, parents and children were similar in their reports of Cohesion and Flexibility (p = 0.72, p = 0.25, respectively). Associations between the problem-solving task and reports of Cohesion and Flexibility were not significant (p = 0.37, p = 0.58, respectively). However, higher agreement among family members on family features was significantly associated with better performance on the task (Cohesion: r = -.76, p < .001; Flexibility: r = -.48, p = .005). These findings underscore the value of integrating both self-report and behavioral assessments to gain a more comprehensive understanding of family functioning in late life.

